# Variations in Facebook Posting Patterns Across Validated Patient Health Conditions: A Prospective Cohort Study

**DOI:** 10.2196/jmir.6486

**Published:** 2017-01-06

**Authors:** Robert J Smith, Patrick Crutchley, H Andrew Schwartz, Lyle Ungar, Frances Shofer, Kevin A Padrez, Raina M Merchant

**Affiliations:** ^1^ Penn Medicine Social Media and Health Innovation Lab Penn Medicine Center for Health Care Innovation University of Pennsylvania Philadelphia, PA United States; ^2^ Positive Psychology Center Department of Psychology University of Pennsylvania Philadelphia, PA United States; ^3^ Department of Computer and Information Science Stony Brook University Stony Brook, NY United States; ^4^ Department of Computer and Information Science University of Pennsylvania Philadelphia, PA United States; ^5^ Department of Emergency Medicine University of Pennsylvania Philadelphia, PA United States

**Keywords:** Facebook, depression, natural language processing, social media

## Abstract

**Background:**

Social media is emerging as an insightful platform for studying health. To develop targeted health interventions involving social media, we sought to identify the patient demographic and disease predictors of frequency of posting on Facebook.

**Objective:**

The aims were to explore the language topics correlated with frequency of social media use across a cohort of social media users within a health care setting, evaluate the differences in the quantity of social media postings across individuals with different disease diagnoses, and determine if patients could accurately predict their own levels of social media engagement.

**Methods:**

Patients seeking care at a single, academic, urban, tertiary care emergency department from March to October 2014 were queried on their willingness to share data from their Facebook accounts and electronic medical records (EMRs). For each participant, the total content of Facebook posts was extracted. Using the latent Dirichlet allocation natural language processing technique, Facebook language topics were correlated with frequency of Facebook use. The mean number of Facebook posts over 6 months prior to enrollment was then compared across validated health outcomes in the sample.

**Results:**

A total of 695 patients consented to provide access to their EMR and social media data. Significantly correlated language topics among participants with the highest quartile of posts contained health terms, such as “cough,” “headaches,” and “insomnia.” When adjusted for demographics, individuals with a history of depression had significantly higher posts (mean 38, 95% CI 28-50) than individuals without a history of depression (mean 22, 95% CI 19-26, *P*=.001). Except for depression, across prevalent health outcomes in the sample (hypertension, diabetes, asthma), there were no significant posting differences between individuals with or without each condition.

**Conclusions:**

High-frequency posters in our sample were more likely to post about health and to have a diagnosis of depression. The direction of causality between depression and social media use requires further evaluation. Our findings suggest that patients with depression may be appropriate targets for health-related interventions on social media.

## Introduction

More than two billion individuals worldwide have social media accounts [1]. Many, including 71% of adult Internet users in the United States, have a Facebook account, and 70% of users report utilizing the platform on a daily basis [2]. The digital divide is also narrowing because social media users are increasingly diverse, representing all strata of gender, race, age, income, and education [2]. Although social media is still evolving, the scope of human engagement with social media tools is vast, suggesting that these tools may be used to glean meaningful insights about human health and behavior. For example, prior work has shown that language used on social media can be used to predict county-level heart disease, track public sentiment around vaccines, and predict disease outbreak [3-6]. To identify the patient groups and disease entities most appropriate for targeting via social media interventions, we sought to examine differences in posting quantity on social media across health conditions.

In a given year, patients may only spend a few hours with clinicians in a physical, face-to-face context [7]. This amount of contact is limited in comparison to the vast majority of time that patients spend outside the confines of the doctor’s office. The concept of “automated hovering” proposes the development of initiatives to follow patients’ routine, everyday behaviors (eg, diet, exercise, and medication adherence) in a manner that is welcomed and convenient for patients for the purpose of improving health outcomes [7]. Given that Facebook’s daily active user base is more than a billion people worldwide, this presents an opportunity to consider the potential for health intervention in the social media sphere [8].

Within public health, social media platforms are increasingly being explored as an avenue for health-related interventions. These interventions have included using Twitter to support smoking cessation efforts, Facebook to encourage physical activity in college students, and online forums to enhance emotional support among cancer patients [9-11]. Less focus has been on which diseases and which cohorts of patients will be the most receptive to social media interventions. Patients within disease groups who are already sufficiently engaged with social media may be the most likely to respond to social media interventions. Alternatively, due to the personal use of these tools, health interventions through these platforms may not be welcomed at all.

In this study, we focused on posting frequency and content on a social media platform (Facebook) as one particular measure of social media activity. We sought to (1) describe variability in social media use across a cohort of social media users within a health care setting and explore the language topics correlated with frequency of use, (2) evaluate the differences in the quantity of social media postings across individuals with different disease diagnoses, and (3) determine if patients could accurately predict their own levels of social media engagement to evaluate the ability to use self-report of social media usage as a proxy for actual use. This work has the potential to inform which patient groups may be most accessible for social media interventions.

## Methods

### Study Design

This was a prospective study of patients seeking care in a single, urban, adult emergency department (ED) from March to October 2014. The study was approved by the Institutional Review Board at the University of Pennsylvania. Patients were excluded if they had severe trauma, were younger than 18 years, in respiratory distress, or had evidence of other severe illness. Patients were asked if they used Facebook. If patients responded affirmatively, they were asked about their willingness to participate in a study about social media. They were then told that the study entailed “sharing” data from their Facebook accounts (eg, statuses, photos, likes, demographics) and their electronic medical records (EMRs) with health researchers.

**Figure 1:**
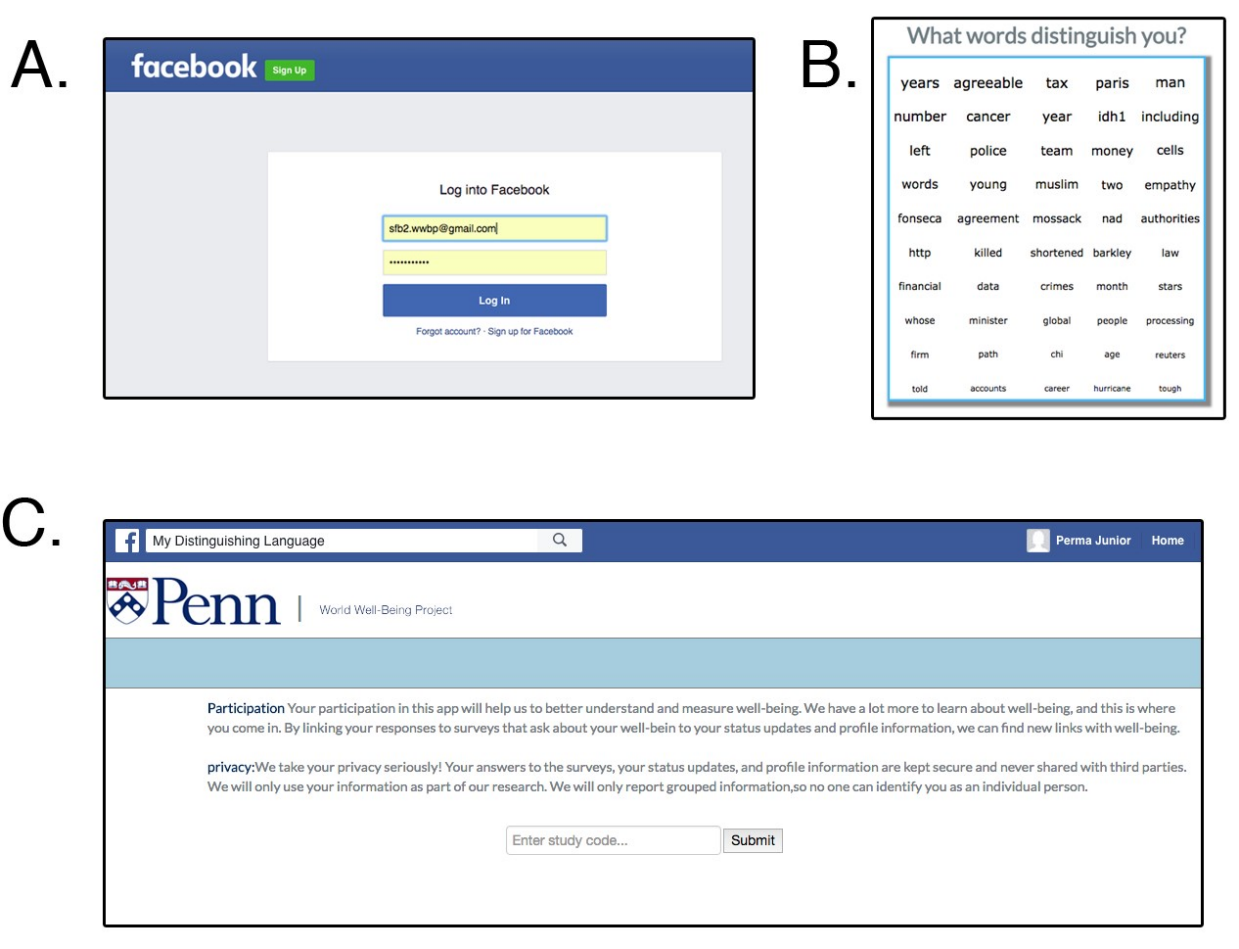
Screenshots of data collection from the Facebook app. (A) illustrates the log-in page for the app, (B) illustrates a language description task for users, and (C) illustrates part of the consent and privacy process for the study.

Participants willing to share their Facebook data underwent a thorough consent procedure. (A thorough analysis of sharer versus nonsharer subsets can be found in Padrez et al [12].) A written consent form was reviewed with a research assistant. A copy of the written consent was then given to the patient. The document explained the types of information that would be extracted from the patient’s social media and EMR data. Patients willing to share their Facebook information were then directed to log in to their Facebook accounts and add a Facebook “plug-in” app related to this study. Before adding the plug-in, another agreement screen appeared detailing the information that would be automatically extracted by the research team. This app was designed for internal research use by the research team using Facebook’s public “Graph” application programming interface (API) with the primary purpose of extracting status updates and limited account information [13]. A secondary function gives feedback to the user about their most frequently used language on Facebook relative to the general population. A screenshot of this Facebook app can be seen in Figure 1. The app only requires a Facebook account and is not tied to a specific browser or operating system.

### Assessing Variability in Social Media Use Across a Cohort

To assess Facebook use across the study cohort, we used an automated process to extract data from each participant’s Facebook account using the previously described Facebook app. We extracted the following variables from each user’s Facebook account: number and content of status updates, and number of friends. For each participant, the total number of Facebook posts was extracted over 6 months prior to study enrollment.

Medical and social media data were stored on servers compliant with the Health Insurance Portability and Accountability Act at the University of Pennsylvania in accordance with protocols approved by the Penn Institutional Review Board.

### Assessing Language Most Commonly Associated With High- and Low-Posting Frequency

To distil the language of our sample into a smaller feature space, we used a natural language processing technique; specifically, we used an unsupervised clustering algorithm, latent Dirichlet allocation (LDA) [14] implemented in the MALLET package [15]. LDA assumes that each document (in our case, each Facebook status post) is a mixture of “topics,” in which each word is produced by one of the unobserved topics. The LDA algorithm automatically finds the maximum likelihood word-topic assignment, and produces topics that are clusters of words that tend to co-occur in documents; these can be days of the week or more abstract groups, such as profanity or words related to food and drink. The parameter for number of topics was chosen prior to any statistical analyses based on visual inspection with the goal of balancing topic coherence (specificity) with coverage of themes used in our Facebook post sample (sensitivity).

### Assessing Variability in Social Media Use Across Disease Diagnoses

Demographic variables (age, race, and sex) were extracted from the patient’s EMR. Using *International Classification of Diseases, Ninth Revision* (*ICD-9*) codes extracted from the EMR, we identified the top eight prevalent conditions in our sample based on each patient’s available historical EMR (years 1997-2014). In addition, during enrollment, patients completed the Patient Health Questionnaire-2 (PHQ-2), a two-question depression screen [16].

### Assessing Accuracy of Perception of Social Media Posting

During enrollment, participants were surveyed about their perceived frequency of posting on social media [2]. Patients were grouped into four categories based on their reported frequency of posting (≥3 times daily, 1-3 times daily, every few days, about once per week or less).

### Statistical Analysis

#### Language and Posting Frequency

**Figure 2:**
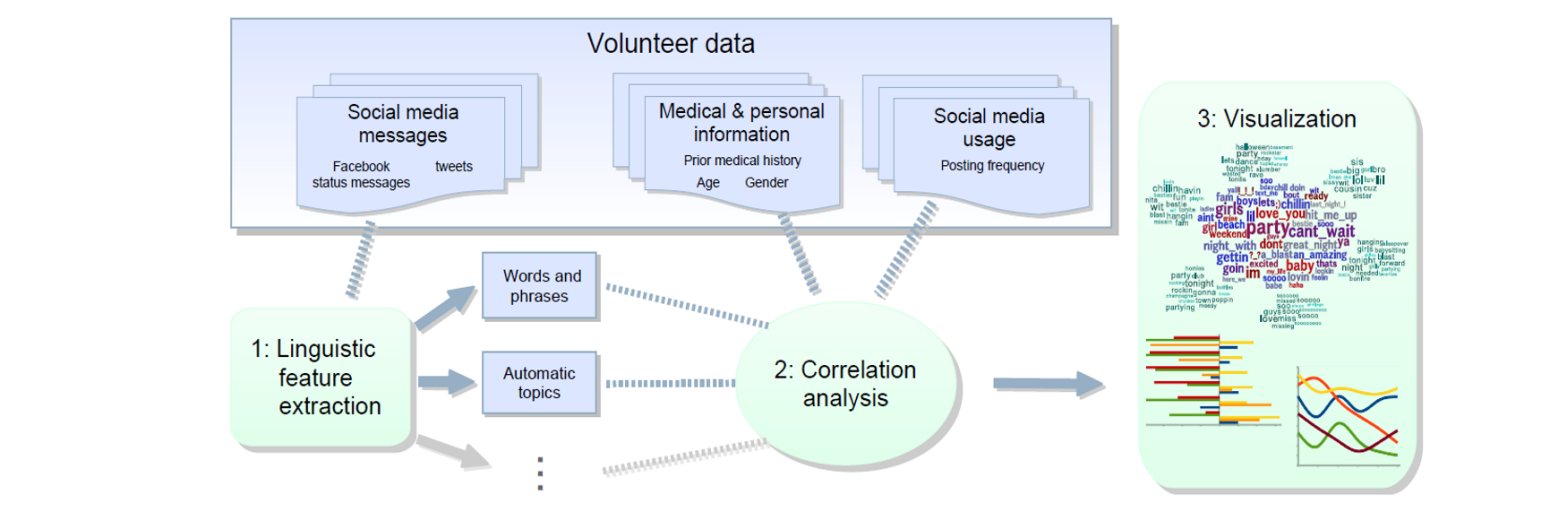
Steps in language correlation analyses (adapted from Schwartz et al [17]). Broadly, step 1 involves deriving independent language variables from the aggregated participant Facebook language data. In step 2, language topics are correlated with other participant characteristics. In step 3, world clouds and other figures are created to visualize the language-characteristic correlations.

After deriving 500 topics, including the probability of each word given each topic, from our sample of Facebook status messages, we applied the topic models to each participant’s complete Facebook posting history, giving us a distribution over the 500 topics for each participant. These made up our independent language variables. Each topic was correlated with a given outcome (eg, membership in the top-posting quartile), controlling for age, sex, and race. To determine the significance of a topic, each language variable (topic) was standardized and separately used as an independent variable in a linear regression to predict each outcome. Linear regression with a binary dependent outcome variable was employed to preserve interpretability of the coefficient; standardized linear regression coefficients are analogous to Pearson product moment correlation. Ordering of results did not change running the same analyses with logistic regression. Control variables (categorical age, race, sex) were binarized (eg, isFemale, isAgeBin1, isAfricanAmerican) and used as additional independent variables to extract the specific predictive power of the language variable. We obtained the standardized regression coefficient for the language variable (hereafter referred to as β), calculated *P* based on degrees of freedom, and then corrected for multiple comparisons (across 500 topics) using the Benjamini-Hochberg method [17]. Statistically significant topics were then presented as clusters of related words associated with posting frequency. For example, we specifically examined age-, race-, and sex-adjusted topics by quartiles of posting frequency. See Schwartz et al [18] for additional details on the language correlation analysis outlined here, as well as Figure 2 for an outline of this process.

#### Posting Frequency and Diagnoses

Because the number of posts for each participant over time was not normally distributed, a log transformation was performed prior to analysis. To compare differences in quantity of Facebook posts across demographics and health conditions, we used the two-sample *t* test for binary variables and one-way ANOVA for variables with three or more categories. To further elaborate differences in posting between individuals with and without each disease condition, we employed an analysis of covariance (ANCOVA) adjusting for the covariates of sex, age, and race. For purposes of presentation, all transformed means are presented as the antilog (ie, data transformed back into their original form). These analyses were performed using SAS statistical software version 9.4 (SAS Institute, Cary, NC, USA).

#### Perception of Social Media Use

The mean number of actual posts was calculated within each category of perceived posting frequency and compared across groups using a one-way ANOVA. Projected number of posts was interpolated for each category based on the description over time (eg, a perception of posting 1-3 times daily over 6 months equates to an estimated total of 180-540 total posts in that time period).

## Results

### Enrollment and Demographics

A total of 1433 patients agreed to participate in a study related to social media and health. Of those, 1008 (70.34%) agreed to share their social media (eg, Facebook) and EMR for this study. Of these, 250 had accounts with inaccessible data. Of the remaining 748 participants, we excluded 53 (7.1%) participants who solely utilized Twitter and did not report having Facebook accounts. Among the 695 Facebook sharers, the mean age was 28.6 (SD 8.9) years, 74.0% (514/695) were female, and 70.4% (489/695) self-identified as African American/black. See Table 1 for statistics on the sample reported here.

**Table 1:** Facebook posts in the 6-month period prior to enrollment and demographics of participants (N=695).

Demographic		n	Mean (95% CI)^a^	*P* value
**Sex**				
	Female	514	27 (24-32)	.006
	Male	181	18 (15-23)	
**Race**				
	African American/black	489	27 (24-32)	.04
	White	141	19 (14-25)	
	Other race	65	20 (13-31)	
**Age (years)**				
	18-29	437	28 (24-33)	.02
	30-39	173	20 (16-26)	
	40-49	60	23 (15-35)	
	>49	25	12 (6-23)	
**Facebook friend count**				
	Q1 (874-4800)	173	40 (31-51)	<.001
	Q2 (483-873)	175	22 (17-28)	
	Q3 (295-482)	175	26 (21-34)	
	Q4 (13-294)	171	16 (12-21)	
**Posting frequency**				
	≥3 times daily	154	51 (40-66)	<.001
	1-3 times daily	161	42 (33-53)	
	Every few days	176	25 (20-32)	
	Once per week or less	204	9 (7-11)	

^a^All analyses were performed using log_10_ of Facebook posts. Values transformed back for presentation purposes.

### Variability in Social Media Use Across a Cohort

The mean number of posts in the previous 6 months was 25 (IQR 7-89), which equates to a mean of one post per week. The mean number of posts in the previous month was 4 (IQR 1-15), which similarly equates to a mean of one post per week. Because mean frequency of posting was consistent across the two time periods (1 month and 6 months prior to enrollment), we present data for the number of posts over the 6-month period prior to study enrollment. Table 1 illustrates the differences in posting frequency across demographics in our sample. Women posted a mean 27 (95% CI 24-32) posts compared to a mean 18 (95% CI 15-23) posts by men (*P*=.006). Participants older than 49 years had fewer mean Facebook posts (mean 12, 95% CI 6-23) compared to younger (age 18-29) participants (mean 28, 95% CI 24-33, *P*=.02). Across racial groups, patients identifying as African American had the highest posting mean (mean 27, 95% CI 24-32, *P*=.04).

### Language of High-Frequency Users

**Figure 3:**
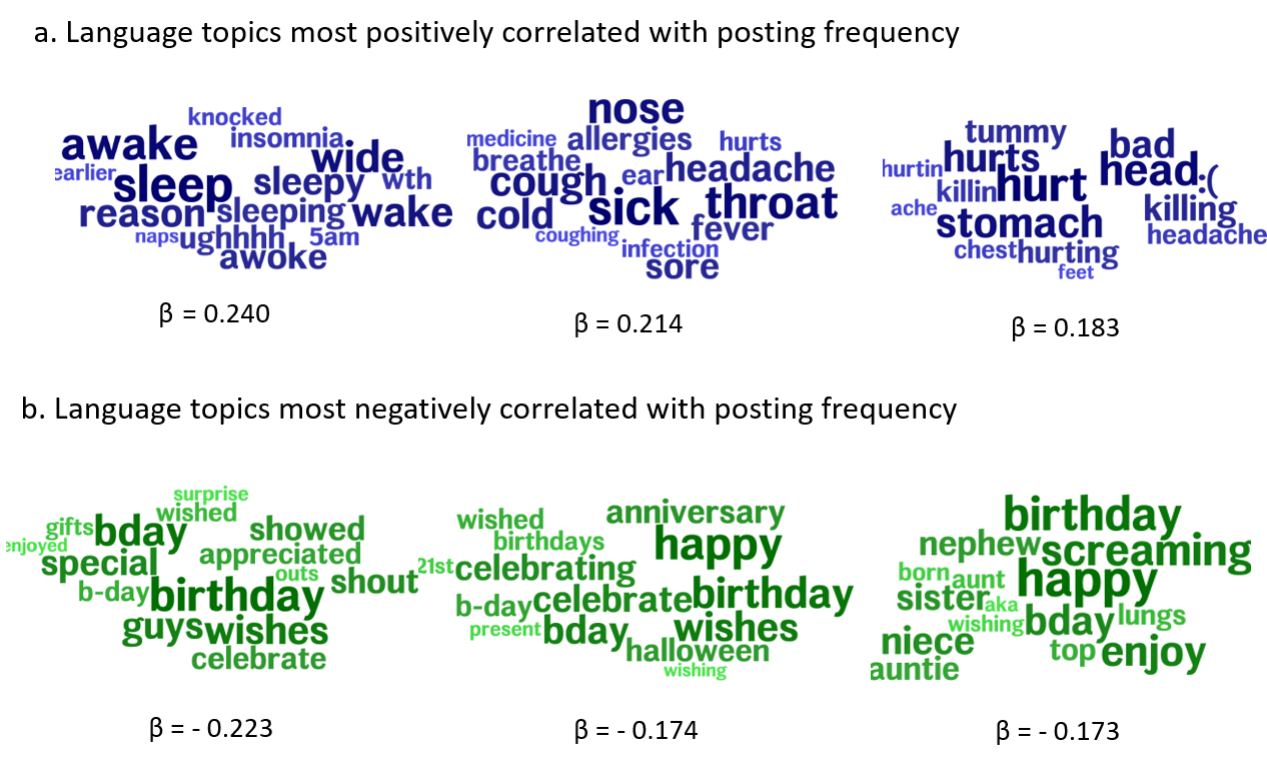
Language of high- and low-frequency Facebook posters. (a) The blue text bubbles illustrate the language topics most positively correlated with participant posting frequency and (b) the green text bubbles illustrate the language topics most negatively correlated with participant posting frequency.

To further characterize the differences in high- and low-frequency users, we developed topic word clouds using LDA. Figure 3 shows that the language topics most highly correlated with being in the highest quartile of Facebook posters (>90 posts in 6 months) related to health and illness. For example, the language topic most correlated with the highest quartile of posters (β=0.240) contained the words “sleep,” “wide,” “sleepy,” “insomnia,” and “wake.” The second-most correlated topic (β=0.214) contained the words “throat,” “sick,” “nose,” “hurt,” and “sore.” The third-most correlated language topic (β=0.183) contained the words “hurt,” “tummy,” “stomach,” “head,” and “bad.” The topics most negatively correlated with posting frequency (β=-0.174) contained language related to “wishes,” “birthday,” “special,” “wishes,” and “celebration.”

### Variability in Social Media Use Across Disease Diagnoses

We examined differences in posting quantity across *ICD-9* health conditions with the highest prevalence (n>50) in our sample (Table 2).

**Table 2:** Prevalence of most common conditions from the electronic medical record within our participant sample (N=695).

Condition	n (%)
Headaches	272 (39.1)
Back pain disorders	243 (35.0)
Anemia	177 (25.5)
Depression	134 (19.3)
Asthma	134 (19.3)
Neoplasm	108 (15.5)
Hypertension	98 (14.1)
Diabetes	66 (9.5)

We also compared posting quantity differences based on the results of the depression screen within the ED. Among patients who screened positive for depression in the ED, the mean number of posts was 38 (95% CI 27-52), which was notably higher than the postings of individuals who did not screen positive for depression (mean 23, 95% CI 20-26, *P*=.003). Individuals with a history of depression or asthma in their past medical history also had higher mean Facebook postings compared to individuals without a history of depression (depression: mean 38, 95% CI 29-50; no depression: mean 22, 95% CI 19-22, *P*=.001) or asthma (asthma: mean 34, 95% CI 24-45; no asthma: mean 25, 95% CI 20-26, *P*=.02). There were no significant differences in Facebook posting frequencies between patients with and without the following conditions: hypertension, diabetes, headaches, back pain, anemia, and cancer. After using an ANCOVA model to control for age, race, and sex, the depression screen and depression medical history were the only health outcomes that showed significant differences in mean posting quantities between patients with and without a disease (Table 3).

**Table 3:** Unadjusted and adjusted (sex, race, age) mean^a^ Facebook posts in the 6 months prior to enrollment by presence or absence of highly prevalent *International Classification of Diseases, Ninth Revision* codes in the electronic medical record.

Health condition		n	Unadjusted mean posts		Adjusted mean posts	
			Mean (95% CI)	*P* value	Mean (95% CI)	*P* value
**Depression screen**						
	Positive	120	38 (27-52)	.003	37 (27-49)	.005
	Negative	575	23 (20-26)		23 (20-26)	
**Depression**					
	Yes	134	38 (29-50)	.001	38 (28-50)	.001
	No	561	22 (19-26)		22 (19-26)	
**Asthma**					
	Yes	134	34 (25-45)	.02	31 (23-41)	.11
	No	561	23 (20-26)		23 (20-27)	
**Headaches**					
	Yes	272	29 (24-35)	.05	28 (23-34)	.69
	No	423	22 (19-26)		23 (19-27)	
**Anemia**					
	Yes	177	23 (18-30)	.57	22 (17-29)	.36
	No	518	25 (22-29)		26 (22-30)	
**Diabetes**					
	Yes	66	26 (17-40)	.73	29 (19-43)	.46
	No	629	25 (21-28)		24 (21-28)	
**Hypertension**					
	Yes	98	26 (18-37)	.78	30 (21-42)	.27
	No	597	25 (21-28)		24 (21-27)	
**Neoplasm**					
	Yes	108	24 (17-34)	.94	26 (19-36)	.69
	No	587	25 (22-28)		24 (21-28)	
**Back pain**					
	Yes	243	25 (20-31)	.96	25 (20-31)	.96
	No	452	25 (21-29)		25 (21-29)	

^a^All analyses were performed using log_10_ of Facebook posts. Values transformed back for presentation purposes.

### Perception of Posting Frequency

Within the sample, 154 of 695 (22.1%) participants reported posting on Facebook three or more times per day, 154 of 695 (22.1%) reported posting one to three times per day, 176 of 695 (25.3%) reported posting once every few days, and 204 of 695 (29.3%) reported posting once a week or less. There was no significant variation in self-reported posting frequency based on gender, race, or age.

**Figure 4:**
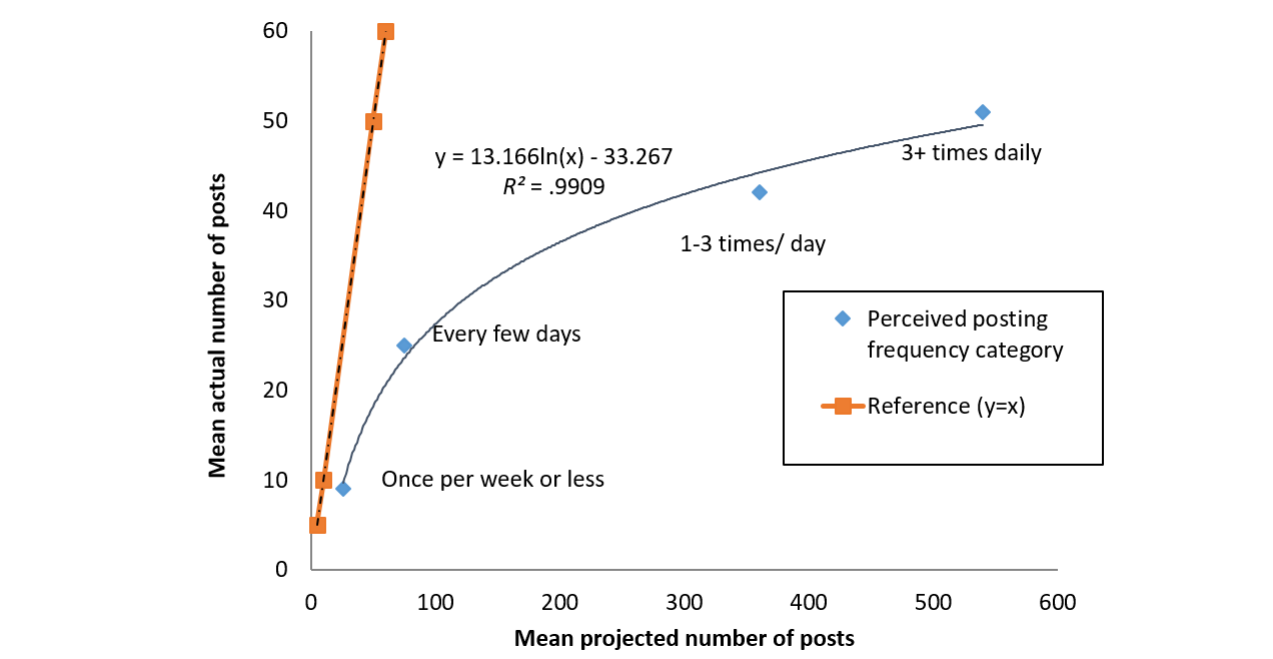
Actual versus projected mean number of posts by perceived posting frequency.

Participants who reported posting on Facebook more frequently did, in fact, have more Facebook posts. For example, in the 6 months prior to enrollment, among participants who reported posting three or more times daily, the mean total posts was 51 (95% CI 40-66) compared to mean 42 (95% CI 33-53) posts in the one to three times daily category, mean 25 (95% CI 20-32) posts in the every few days category, and mean 9 (95% CI 7-11) posts in the once per week or less category. The difference in mean posting frequency across the four posting frequency groups was significant over the 6 months prior to enrollment (*P*<.001). Figure 4 illustrates the logarithmic relationship between mean actual posting amount and mean projected posting amount based on the four categories of perceived posting quantity.

## Discussion

Our study had four major findings: (1) there was significant variation in Facebook posting frequency within our patient sample; (2) high-frequency posters wrote about topics related to health; (3) when controlling for demographic variables, there were significant differences in Facebook posting quantities between individuals who screened positive for and/or had a clinical diagnosis of depression; and (4) patients are relatively good predictors of their Facebook posting frequency over time.

### Variation in Social Media Posting

We found that there was significant variation in Facebook posting frequency within our patient sample. These differences may allow us to identify and distinguish health-related characteristics that are specific to subsets of patients based on their levels of social media use.

### High-Frequency Posters Write About Topics Related to Health

The language topics most correlated with the highest quartile of users in our sample indicate that high-frequency users communicate about health and disease on Facebook profiles. This suggests that there may be further insights to be gained from studying the language of social media within this high-posting population. By contrast, among the low-frequency posters, the topics of “birthday” and “celebration” indicate that this subset of the sample is primarily posting on Facebook in response to being prompted by the Facebook platform (ie, to wish someone a happy birthday). The language of low-frequency posters may be less rich for identifying potential health interventions.

### Depression and High-Posting Frequency

Our study suggests that posting frequency was associated with depression, but not with other disease diagnoses. Individuals screening positive for depression symptoms are more likely to post more frequently on Facebook, regardless of the content of their posts. As such, depression and other mental health conditions may be ideal candidates for pursuing social media interventions. We know that social media platforms are being mined for insights about health and disease with the potential for meaningful interventions [19,20]; in fact, Facebook recently introduced an online tool to help identify individuals at high risk for suicide [21].

There could be several explanations for our finding that high-frequency posting seemed to be more common in those with positive depression symptoms or a diagnosis of depression. In fact, our results provide an extension to a growing body of literature on this topic. Previous work by Bessière et al [22] showed that increased use of the Internet for the purpose of connecting with friends and family is associated with decreased rates of depression. However, work by Sagioglou and Greitemeyer [23] showed that increased Facebook usage, although distinct from total Internet use, correlated with negative mood. In contrast, other reports showed no association between time on social networking sites and PHQ-9 score [24]. Work by Hampton et al [25] suggested that high-frequency exposure to digital media may cause users to be more aware of stressful events in the lives of others, indirectly causing higher stress levels in the user, termed the “cost of caring” [26]. Other work by Steers et al [27] suggests that increased time on Facebook causes people to feel depressed due to comparative feelings of inadequacy. Our work is distinct within this growing body of literature in that we used a validated EMR, as opposed to self-report, to identify patient mental health conditions.

It is unclear at this point what direction the depression-posting relationship entails. Individuals in the developing stages of depression may be posting on Facebook with greater frequency as a means to reach out to a social network, escape a sense of isolation, or maintain connectivity. Conversely, it is possible that high-frequency activity on Facebook or any other virtual connectivity platform may contribute to underlying depression. Either way, knowing that a user is a high-frequency poster may be a useful signal to indicate appropriate screening for depression.

### Patients Predict Their Own Social Media Use

Accuracy of patient self-reported activity is often either unreliable or unknown in other domains of medicine [28-30]. If social media usage is ever deemed to be a risk factor for the development or presentation of an illness, our findings suggest that patients may provide reliable information regarding their own usage.

### Limitations

This study has several limitations. Findings are reported from a convenience sample of primarily young, predominantly black women who enrolled via a single, urban, academic medical ED. These demographics are not nationally representative, but our sample did represent a target population with high rates of chronic disease. We also limited our analyses to Facebook, although we recognize that social media encompasses a broad range of ever-evolving online platforms. We also recognize that “posting” is merely one proxy of social media engagement because most people silently observe online activity without tangibly contributing, which is harder to observe and track [31].

### Conclusion

Posting frequency on Facebook varied across demographics and health conditions. In our sample, individuals with depression were more likely to post more content on Facebook than those who were not depressed, suggesting that depression may be an ideal disease target for intervention on social media. Furthermore, the language of high-frequency posters illustrates that patients do post about health. Patients are relatively accurate assessors of their own social media posting habits.

## Abbreviations

API: application programming interface
ED: emergency department
EMR: electronic medical record
ICD-9: International Classification of Diseases, Ninth Revision
LDA: latent Dirichlet allocation
PHQ: Patient Health Questionnaire
